# Is the oral health reform in Israel optimally distributed? - A commentary

**DOI:** 10.1186/s13584-019-0302-z

**Published:** 2019-03-20

**Authors:** Harold Sgan-Cohen, Guy Tobias, Avraham Zini

**Affiliations:** Hebrew University-Hadassah Faculty of Dental Medicine, POB 12272, 91120 Jerusalem, Israel

**Keywords:** Dental public health, Health policy, Health care, Dental health care, National health insurance law

## Abstract

A traditional and ethical principle recognizes a country’s primary general welfare responsibility to the young and the old. However, the middle, adult, age group cannot and should not be disregarded. The current dental component of the National Health Insurance Law (NHIL), in Israel, only includes children and the elderly. The present commentary focuses on the large group of adults, age 19–74, which are currently excluded.

The cumulative incidence of disease increases over the lifetime of a person. We believe that a NHIL commitment with a major age gap in coverage is unacceptable. The recent manuscript, published by Natapov et al., in this journal, has documented the overall dental health of the older Israeli population, with emphasis on nutritional aspects. This contribution to the literature is commendable. However, we aim to follow in the steps of the Alma Ata Declaration and Ottawa Charter of the World Health Organization (WHO) and to clarify that the government’s responsibility should cover all residents regardless of their age. In addition, a dental health epidemiological data base, currently nonexistent for adults, is called for.

The National Health Insurance Law (NHIL) of 1995 [1] ensures basic health coverage for every resident of the country and defines the government’s accountability to provide optimal health services to all persons without discrimination. According to the law: “Medical insurance… shall be based on principles of justice, equality and mutual assistance”. All residents have the right to register as a member of one of four Health Maintenance Organizations (HMOs) of his/her choice, free of any preconditions or limitations stemming from his/her age or other variables associated with the state of his/her health.

A recent addendum to the NHIL now includes 16–18 year olds, thus covering all children, ages 0–18. Elderly people, over 75 years of age, are now expected to be granted basic oral health care services in the near future.

Representatives of the Israel Health Ministry (Natapov et al) have published a manuscript describing the dental status, visits, functional ability and dietary intake of the elderly in Israel. [2] The article presents a broad depiction of dental health and its determinants among Israeli elderly, and it argues that including this age segment of the population is indicated and essential.

The World Health Organization (WHO) Alma Atta Declaration of 1978 describes the essential value of “Health for All” [3]. The WHO Ottawa Charter of 1986 declared that “Health promotion action aims at reducing differences in current health status and ensuring equal opportunities and resources to enable all people to achieve their fullest health potential” [4]. It should be noted that “all people” are mentioned without any limitation to age.

We do not challenge the special right of treating the young and the old. The vast majority of health and disease including oral health is a life course continuum. It commences and originates among the younger age groups. The elderly have unique needs, related often to low socio-economic status and prolonged chronic complications of pre-existing pathologies [5–7]. We have no intention to ignore the overriding moral ethic in Israel, and many other countries, which indicates taking care of the elderly.

Oral diseases are chronic, debilitating, cumulative and increasingly severe with age [8]. This escalation occurs throughout life, without exclusion of the years of adulthood (ages 19–74), which are currently ignored in the Israeli NHIL.

For example, periodontal (gum) disease usually evolves after inferior oral hygiene and development of dental caries (cavities). It emanates from a sweet diet among other factors [9]. Both of these major oral pathologies, caries and periodontal disease, originate in early childhood, as do their related health behaviors. Later on in life, health behavior habits are exceedingly difficult to change.

The epidemiological literature has clearly demonstrated a strong association between dental and general health [10]. This includes the relationship between caries and periodontal (gum) disease and obesity, chronic heart disease, diabetes, pulmonary infection, a wide range of conditions, including septicemia, mental pathologies, premature childbirth, and more. A “Common Risk Factors” approach has been advocated. [11] This include factors such as smoking, excessive sweet diet, and others. As examples, smoking causes a wide range of medical diseases, but also periodontal disease; sweet food consumption causes diabetes and obesity, but also dental caries. The large proportion of these common risks, and their consequences, involve adulthood.

Originally, the Israeli NHIL was based upon the principle of health for all, but it did not include any dental health component until 2010. Then, the addition focused on younger age groups, and not on the entire population [12].

Unfortunately, a large-scale survey focusing on middle aged adults’ dental status and needs has never been conducted in Israel. This reflects the inadequate attention to the adult age group, concerning dental health.

While the early age groups can be regarded as of paramount importance, ages 18–75 years, the ages that account for the majority of the population, cannot be ignored. This middle age group contributes the major work and financial contribution to the strength, stability and growth of the economy. Younger adults, experiencing inferior dental care, often due to problems of high costs and inaccessibility, very likely will be faced with complications of edentulousness (lack of all or most teeth). This, in turn, will place an even heavier weight on the health care system.

The bulk of dental care is delivered to adults. This includes a significant price tab that includes issues of work hours lost, adequate supply of dentists, hygienists and dental technicians, and a significant influence on quality of life.

An obvious question is what the price tag of our proposal would be. In any location, governments are faced with a budget priority dilemma. Israel is a small country with a wide range of national security and welfare needs. The government shareholders are obligated to justify each and every extra cost.

The present commentary has not calculated the extra cost of covering middle aged dental needs. Although in the past, changes in the dental health package have been based solely on political and social considerations, since we believe that it is important to provide dental coverage and care to the entire adult population, we also believe it is important to evaluate the implications, including economic implications, of doing so and would urge the government with its responsibility for the overall health of the population to do this.

## Conclusion

The basic rationale and philosophy of the Israeli NHIL does not mention any age limitations. Dentistry has always been the exception to the rule, however the present age gap from 19 to 74 cannot be justified on a scientific, epidemiologic, health, or ethical basis.

## Abbreviations

HMO: Health Maintenance Organization
NHIL: National Health Insurance Law
WHO: World Health Organization
